# Control of Transcriptional Elongation by RNA Polymerase II: A Retrospective

**DOI:** 10.1155/2012/170173

**Published:** 2012-01-29

**Authors:** Kris Brannan, David L. Bentley

**Affiliations:** Department of Biochemistry and Molecular Genetics, University of Colorado School of Medicine, Aurora, CO 80045, USA

## Abstract

The origins of our current understanding of control of transcription elongation lie in pioneering experiments that mapped RNA polymerase II on viral and cellular genes. These studies first uncovered the surprising excess of polymerase molecules that we now know to be situated at the at the 5′ ends of most genes in multicellular organisms. The pileup of pol II near transcription start sites reflects a ubiquitous bottle-neck that limits elongation right at the start of the transcription elongation. Subsequent seminal work identified conserved protein factors that positively and negatively control the flux of polymerase through this bottle-neck, and make a major contribution to control of gene expression.

## 1. Introduction

The initiation phase of the RNA polymerase II (pol II) transcription cycle involves multiple events, including recruitment of general transcription factors and pol II to the promoter, melting of the DNA template, initiation of RNA synthesis, and pol II promoter clearance, which marks entry into the elongation phase. The stochastic nature of all of these steps poses a potential problem if it becomes necessary to mount a rapid activation of transcription. Following initiation pol II often encounters a rate-limiting barrier that appears to lie between early elongation and productive elongation. The transition between these two phases of the transcription cycle has now been characterized as a powerful regulatory switch used to increase or decrease gene expression in a signal-responsive fashion. Here we review the early discoveries that laid the foundation for a detailed understanding of transcriptional regulation at this transition.

## 2. Early Evidence of Polymerase Pausing and Premature Termination in DNA Viruses

Nearly 30 years ago it was reported by the late Yosef Aloni and colleagues that run-on transcripts made in nuclei from SV40 infected cells were strongly biased toward the 5′ end of the late transcription unit suggesting that pol II accumulated in the promoter-proximal region [1]. Analysis of labeled RNA extended on viral transcription complexes (VTCs) assembled *in vivo* and purified from infected cells revealed two additional unusual features of transcription from the late promoter. First, two pause sites were mapped around positions +15 and +40 relative to the start site by identifying the junctions between unlabelled RNA made *in vivo* and labeled RNA extended *in vitro* [2]. Second, a major product of transcription on VTCs is a discrete 93–95 base RNA, that is, prematurely terminated near a potential hairpin loop structure. Similar evidence for promoter-proximal stalling and/or premature termination were subsequently reported for the early and late promoters of polyoma virus [3]. These results prompted speculation that SV40 late transcription might be regulated by a mechanism [1] that regulates a decision between premature termination and productive elongation, analogous to attenuation on bacterial operons [4]. About the same time Luse and colleagues showed that transcription complexes assembled in HeLa nuclear extract on the adenovirus 2 major late promoters under NTP limiting conditions gave rise to uncapped transcripts about 20 nucleotides long that could be elongated into capped transcripts upon NTP addition [5]. The implication of this result is that pol II can pause at relatively discrete positions near the transcription start site and remain competent to resume elongation. They called this phenomenon “promoter-proximal pausing.” Together these seminal early studies revealed quite unexpected patterns of stalling, pausing, and premature termination by host cell's pol II when it transcribes certain viral genes. The question posed by these studies was whether this unusual behavior by pol II was peculiar to viral genes or shared in common with cellular genes.

## 3. Pol II Pile-Ups on Cellular Genes

**Figure 1:**
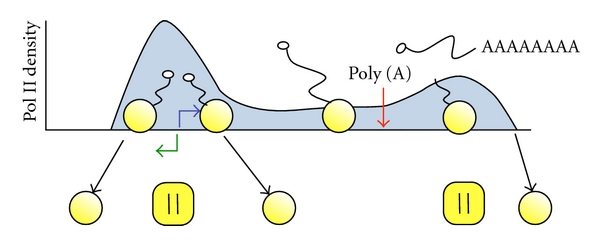
RNA pol II density profile across a typical metazoan protein-coding gene. Elevated density around the transcription start site (TSS) results from promoter-proximal pausing and possibly premature termination of transcription. Blue and green arrows denote divergent transcription from the TSS. A second peak of pol II accumulation downstream of the poly (A) site precedes termination coupled to cleavage/polyadenylation. Black arrows denote termination of transcription with eviction of pol II (yellow circles) from the DNA template downstream of the poly (A) site (red arrow) and possibly also in the promoter-proximal region. The mRNA cap structure is denoted by a white circle.

It was not long before the first evidence emerged that pol II also piles up near the transcription start sites of cellular genes. High levels of pol II were found to accumulate at the 5′ ends of the Drosophila heat shock gene *hsp70* [6, 7], and human *c-myc* genes even though the genes were not actively expressed [8, 9]. These 5′ polymerases were not only able to incorporate labeled NTPs in the nuclear run-on reaction but were also resistant to sarkosyl. Moreover, in some cases they were demonstrated to be associated with a single-stranded transcription bubble showing definitively that they were actively engaged on the template [10]. Subsequent run-on studies revealed that pol II was distributed with a similar strong bias in favor of the promoter-proximal region on Hsp26 and GAPDH in Drosophila [11] and adenosine deaminase, c-fos, DHFR and transthyretin genes in mammals [12–15]. As a footnote several of these early nuclear runon studies detected transcription proceeding in both directions from the start site, but the significance of this divergent transcription remained obscure [8, 16]. These results therefore showed that the pattern of pol II accumulation near start sites, first observed in DNA viruses, was common to a number of cellular genes. In fact it emerged from these early studies that pol II accumulated near the TSS of most or all cellular genes where it was localized in sufficient detail. Based on this evidence Krumm and colleagues suggested in 1995 that promoter-proximal pausing was a “general rate-limiting step” in the pol II transcription cycle [17]. Recently, this prediction has been largely borne out by ChIP-seq and Gro-Seq studies that localized pol II genome-wide and found high levels of pol II accumulation at the start sites of thousands of genes in Drosophila and human cells [18–20]. Indeed in human cell lines relatively few genes have a uniform distribution of pol II throughout their length compared to those with a promoter-proximal pol II pile-up (H. Kim, S. Kim, K. Brannan and D. Bentley unpublished observations). Promoter-proximal pol II accumulation likely involves sequence elements upstream and downstream of the start site as well as chromatin structure [21–23]. While the details of what makes pol II pile-up near start sites remain somewhat obscure, this is clearly a characteristic shared by numerous promoters (Figure 1). 

## 4. Promoter-Proximal Pausing versus Premature Termination

What is the root cause for why pol II is so unevenly distributed across so many genes? The original *in vitro* pulse chase experiments of Coppola and colleagues showed that pol II can pause close to the start site and then resume elongation [5]. Since then, the most popular interpretation of *in vivo* polymerase mapping studies has been that they result from a similar “promoter-proximal pausing” phenomenon. That at least some promoter-proximal polymerase can resume elongation is demonstrated by nuclear runon experiments; indeed, these polymerases would not be detectable by this method if they could not elongate and incorporate labeled nucleotides. However, the possibility that some fraction of the promoter-proximal polymerases terminate prematurely and never enter the productive elongation phase cannot be eliminated. The evidence for premature termination is quite clear for the SV40 late and HIV viral genes [24, 25], but it is much less compelling for cellular genes. Prematurely terminated RNAs are a major product of *c-myc* transcription in microinjected Xenopus oocytes, but the physiological relevance of this phenomenon remains unproven [26]. Recently, short (20–90 bases) transcription start site-associated (TSS-a) sense and antisense transcripts present at very low levels in the nucleus were detected by high-throughput RNA sequencing [27]. Whether these transcripts are products of promoter-proximal premature termination or pol II pausing are interesting questions for future investigation.

## 5. The Function of Polymerase Accumulation at Start Sites

An important question to emerge from the early studies of pol II localization on viral and cellular genes was: “What is the purpose of pol II piling up at the start sites of genes even before they are activated?” One answer to this question quickly emerged from studies of three genes with regulated transcriptional output: the cellular *Hsp70* and *c-myc* genes [6, 8, 9] and a transfected reporter driven by the HIV1 LTR [24]. In each of these cases nuclear run-on transcription revealed a key difference between the activated and nonactivated states: the ratio of polymerases within the gene body relative to the 5′ end increased when transcription was activated. The significance of these studies is that they showed regulation of gene expression can be exerted at the level of transcriptional elongation by controlling the fraction of polymerases that are permitted to travel beyond the promoter-proximal region. Furthermore at *Hsp70*, the amount of paused pol II prior to heat shock correlated with the amount of mRNA made after heat shock [23]. Therefore, a satisfying answer to the question of why pol II accumulates near start sites is that it provides a pool of engaged polymerases ready for rapid mobilization in response to a gene activation stimulus. A second way that localized pol II accumulation at the TSS may enhance rapid transcriptional responses is excluding nucleosomes, thereby providing a bookmark in the chromatin that can be easily accessed by the transcriptional machinery [22]. A third suggestion is that an extended pol II dwell time within the promoter proximal region allows for cotranscriptional capping of the nascent mRNA [28, 29], and could help to “license” productive elongation complexes by allowing time for recruitment of processing and elongation factors. On the other hand, there is no direct evidence that a pol II pile-up near the TSS is required for efficient capping.

## 6. Control of Elongation by Transcriptional Activators

How is the flux of pol II from the promoter-proximal region into the body of a gene controlled? The first important clue was again provided by a virus; in this case HIV1. Groundbreaking work of Kao and colleagues showed that the viral transactivator protein Tat had the novel ability stimulate elongation by pol II [24]. Without Tat, most polymerases that initiate from the HIV1 LTR terminate prematurely shortly downstream of the TAR hairpin loop sequence in a manner resembling the SV40 late transcription unit, but in the presence of Tat, pol II acquires the ability to extend transcripts all the way to the end of the provirus. To explain these surprising results, Kao et al. suggested that Tat regulates transcription by an antitermination mechanism similar to that exerted by the bacteriophage lambda N protein [30]. However, it remained possible that Tat also controlled transcriptional pausing, which is frequently a pre-requisite for termination.

HIV Tat is an unusual transactivator because it binds to the nascent RNA transcript. Therefore, the question remained open as to whether conventional DNA-bound activators can influence transcriptional elongation. Part of the answer to this question came with the demonstration that Tat could activate transcription when tethered to a DNA-binding site in the promoter [31]. Subsequent studies showed that enhancers and promoter-bound chimeric transcription factors comprising activation domains fused to a DNA-binding domain can stimulate elongation [32]. Furthermore a number of natural cellular activators stimulate elongation including heat-shock factor, NFkB, and *c-myc* [21, 33, 34]. Activation domains that enhance elongation and initiation, respectively, can synergize with one another and the most potent activation domains such as Herpes virus VP16 can stimulate both initiation and elongation [35, 36].

## 7. The Yin and Yang of Elongational Control

How do activators like HIV Tat and cellular transcription factors stimulate pol II transit away from the promoter-proximal region and into the downstream region of the gene for productive mRNA synthesis? The solution to this problem was provided by landmark studies that uncovered novel inhibitors of elongation and the factors that antagonize them. This story started with an early insight into how the ATP analogue 5, 6-dichloro-1-ß-D-ribofuranosylbenzimidazole (DRB) inhibits pol II transcription. Pulse labeling of RNA in adenovirus-infected cells revealed that DRB inhibited chain elongation but not initiation [37]. In a *tour de force* of classical biochemistry, the Handa and Price labs took advantage of this inhibitor to identify the core negative and positive factors that control the “yin and yang” of transcriptional elongation. Handa's lab identified the DRB-sensitivity-inducing factor (DSIF) as Spt4/5 a conserved pol II binding complex that is required for inhibition of elongation near 5′ ends [38]. Soon afterwards, these workers identified a second negative-elongation factor, NELF, that cooperates with DSIF [39]. The counterpart to these negative factors is positive transcription elongation factor b (PTEFb) discovered by Marshall and Price [40]. PTEFb was identified as the cyclin-dependent protein kinase complex Cdk9-CyclinT1 [41, 42] that is specifically inhibited by DRB. In a remarkable convergence of independent studies, it turned out that the negative-factors DSIF and NELF and the positive-factor PTEFb are all components of the same control system. Thus, a major function of PTEFb is to “alleviate” the negative effects of DSIF and NELF [43] which it does by phosphorylating them both as well as the pol II C-terminal domain [44, 45].

Elucidation of the interplay between positive- and negative-elongation factors provided a basis for understanding how transcription factors can regulate elongation. The vital missing piece of the puzzle was filled in with the discovery that Tat when bound to TAR in the nascent HIV1 transcript contacts PTEFb through Cyclin T1 and this interaction is required for stimulation of transcriptional elongation [41, 42, 46]. Tat-mediated recruitment of PTEFb permits modification of the paused pol II complex by phosphorylation of the pol II CTD, Spt5, and NELF resulting in a transition to productive elongation. A similar mechanism involving PTEFb-mediated antagonism of the negative-elongation factors DSIF and NELF is thought to regulate elongation at many cellular genes including *c-fos* and *NFkB* targets [45, 47]. PTEFb (Cdk9/CyclinT1) is found embedded in multiple complexes with different protein and RNA subunits [48, 49] and there are likely to be multiple ways that it can be recruited to genes. These include binding directly to transcription factors [33] and chromatin components [50].

## 8. Concluding Remarks

Tremendous advances have been made in understanding control of gene expression at the level of transcriptional elongation since the early days when it was identified on a few viral and cellular genes. Now this mechanism is recognized to be at least as important as control of the initiation step in pol II transcription. Still, important questions remain unresolved about the nature of promoter-proximally accumulated pol II. It is still not clear how many of these paused polymerases have backtracked and are destined ultimately to resume elongation and how many are destined for premature termination. These scenarios suggest the possibility of distinct targets for regulation by controlled polymerase release into the body of the gene. It will be interesting to see how these targets might be used in various developmental and signal-responsive contexts.
